# Emerging Insights into Memory Natural Killer Cells and Clinical Applications

**DOI:** 10.3390/v16111746

**Published:** 2024-11-07

**Authors:** Jonida Kokiçi, Anucha Preechanukul, Helena Arellano-Ballestero, Frances Gorou, Dimitra Peppa

**Affiliations:** 1 Division of Infection and Immunity, University College London, London NW3 2PP, UK; 2 Cancer Institute, University College London, London WC1E 6DD, UK

**Keywords:** natural killer (NK) cells, memory, adaptive NK cells, cytokine-induced memory-like (CIML), HCMV, immunotherapy

## Abstract

Natural killer (NK) cells are innate lymphocytes that can rapidly mount a response to their targets by employing diverse mechanisms. Due to their functional attributes, NK cells have been implicated in anti-viral and anti-tumour immune responses. Although traditionally known to mount non-specific, rapid immune responses, in recent years, the notion of memory NK cells with adaptive features has gained more recognition. Memory NK cells emerge in response to different stimuli, such as viral antigens and specific cytokine combinations. They form distinct populations, accompanied by transcriptional, epigenetic and metabolic reprogramming, resulting in unique phenotypic and functional attributes. Several clinical trials are testing the efficacy of memory NK cells due to their enhanced functionality, bioenergetic profile and persistence in vivo. The therapeutic potential of NK cells is being harnessed in viral infections, with wider applications in the cancer field. In this review, we summarise the current state of research on the generation of memory NK cells, along with their clinical applications in viral infection and cancer.

## 1. Introduction

Natural killer (NK) cells were first described in 1975 for their ability to mount early, rapid and non-specific responses to tumour cells [1].

**Figure 1:**
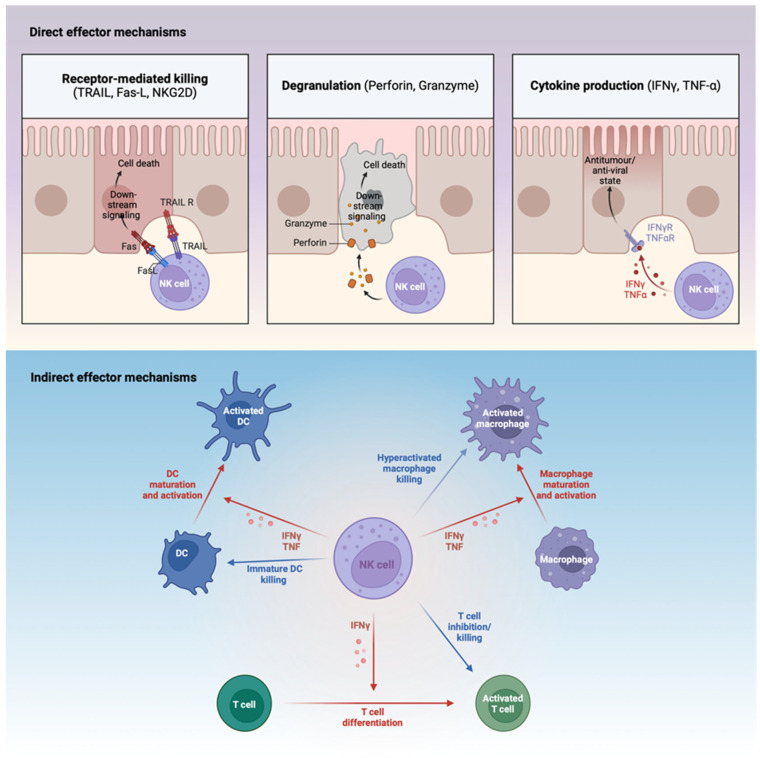
Antiviral mechanisms employed by NK cells. NK cells respond directly by degranulation, receptor-mediated lysis of infected cells and by producing antiviral cytokines like IFNγ in response to target cells. NK cells promote dendritic cell (DC) and macrophage maturation and activation and guide immature helper T cells (Th0) towards an inflammatory phenotype (Th1) via both cytokines cell surface receptors, thus indirectly supporting the adaptive immune response (indicated by red arrows). NK cells also produce chemokines that recruit other immune cells to inflammatory sites. Inversely, NK cells can eliminate (indicated by blue arrows) activated T cells, immature DCs, and overactive macrophages. Fas-L, Fas ligand; TRAIL, TNF-related apoptosis-inducing ligand; TNFα, tumour necrosis factor α; IFNγ-R, interferon-γ receptor.

Due to their diverse effector functions, NK cells have been implicated in anti-tumour and anti-viral immunity. They can lyse their target cells through direct contact via Fas–FasL or TRAIL–TRAILR interactions and the release of lytic granules, such as granzyme and mechanisms involving perforin [2]. NK cells express Fc receptors, which can recognize antibody-coated target cells towards which they degranulate and produce cytokines as part of their antibody-dependent cellular cytotoxicity (ADCC) response [2]. NK cells produce chemokines and cytokines, in particular interferon gamma (IFNγ), which not only has a direct anti-viral role itself [3], but also contributes to the orchestration of robust immune responses. The activation of NK cells is contingent upon the integration of signals from a variety of inhibitory receptors, such as killer cell immunoglobulin-like receptors (KIRs), NKG2A, TIGIT and KLRG-1, and activating receptors, such as CD16, NKG2C, NKp46, NKG2D and 2B4 expressed on their cell surface [2]. Inhibitory receptors like KIRs identify MHC class I molecules present on healthy cells, designating them as “self” and functioning as a regulatory mechanism against NK cell cytotoxicity. This intricate balance maintains self-tolerance while allowing vigorous responses to virally infected cells that may have downregulated their MHC class I molecules [2]. Additionally, these infected cells may upregulate stress ligands or viral-associated molecules recognized by activating receptors such as NKG2D and natural cytotoxicity receptors (NCRs) [4]. In addition to providing help to the immune system by promoting T cell differentiation, DC and macrophage maturation and activation through IFNγ release [5,6,7], NK cells can directly and indirectly inhibit innate and adaptive immune responses, as a means of maintaining homeostasis [8]. For instance, they can eliminate activated T cells, thus hampering the T cell arm of immunity [8,9], and they can indirectly regulate humoral responses through elimination of follicular T helper (Tfh) cells [10]. Similarly, NK can eliminate immature DC cells and overactivated macrophages [11,12]. The close crosstalk between NK cells and other immune cells ensures optimal activation and robust immune responses, while maintaining homeostasis. Figure 1 below summarises key NK effector functions.

Compelling evidence has led to a series of discoveries demonstrating that some NK cell populations lie at the interface of innate and adaptive immunity. These memory-like NK cell populations display characteristics typically associated with adaptive immunity, including clonal-like proliferation of antigen-specific populations exhibiting heightened recall response upon a secondary encounter, which does not involve somatically rearranged antigen-specific receptors [13]. Memory NK cells have been shown to expand in response to various stimuli, including viral antigens and cytokines.

## 2. Antigen-Specific Memory NK Cells and Functional Responses: Lessons from Viral Infections

Antigen-specific memory NK cell responses were first demonstrated in a mouse model lacking antigen-specific memory B cell and T cell responses in contact hypersensitivity reactions to chemical haptens (oxazolone and 2,4-dinitrofluorobenzene) and viral antigens from Human Immunodeficiency Virus (HIV), influenza and vesicular stomatitis virus (VSV) [14,15]. Rag2^−/−^ mice deficient in functional B and T cells challenged with a specific hapten and viral antigens developed an NK-cell-dependent increase in ear swelling upon subsequent exposure to the corresponding haptens and viral antigens, which persisted for at least four weeks. These findings suggest that NK cells play a crucial role in mediating long-lasting, antigen-specific memory responses in the absence of traditional adaptive immune cells [15].

Subsequent research has shown antigen-specific NK cell memory in response to mouse cytomegalovirus (MCMV) infection [16]. Through the engagement of activating receptor Ly49H, NK cells recognise the MCMV-encoded glycophosphatidylinositol-anchored protein m157, which has structural homology to MHC class I [17,18]. When NK cells encounter cells expressing the m157 protein, they trigger NFAT-driven transcriptional activity via the Ly49H receptor, resulting in the swift destruction of the m157-expressing target cells [17,18]. m157 recognition enables a rapid clonal-like expansion of antigen-specific Ly49H^+^ NK, which provides better protective responses against MCMV re-exposure than an equivalent frequency of naive NK cells, akin to antigen recognition by memory CD8 T cells [16,19]. Interestingly, MCMV-specific memory NK cells have diminished clonal proliferation in response to heterologous infection (e.g., Influenza and Listeria) and are less reactive to bystander proinflammatory cytokine stimulation [20], unlike memory CD8 T cells that gain more responsiveness to proinflammatory cytokine stimulation [21].

Clonal analyses of murine NK cells have elucidated that within the virus-specific population, Ly49H^+^ NK cells with high avidity for MCMV peptides clonally proliferated and became enriched with higher expression of the surface receptor Ly49H during both effector and memory responses to MCMV infection [22,23]. This suggests a selective pressure and avidity selection of antigen-specific memory NK cells that can mount a more potent response upon re-encounter in contrast to the classical phenomenon of gene rearrangement that creates antigen-specific receptors in B and T cells.

Analogous to the antigen-specific NK cells in MCMV infection, human NK cells expressing NKG2C and CD57 with adaptive features have been reported in human cytomegalovirus (HCMV) infection [24]. HCMV infection drives an adaptive diversification of NK cells, which form distinct populations, accompanied by transcriptional, epigenetic and metabolic reprogramming resulting in unique phenotypic and functional attributes. In particular, adaptive NK cells are characterised by a high expression of the activating receptor NKG2C, which serves as their identifying hallmark and is structurally homologous to the murine Ly49H [24,25]. Adaptive NK cell populations recognise HCMV-derived peptides presented by HLA-E, providing evidence of antigen-specific human NK cell responses [25]. Characterisation of the peptide repertoire involved in the NKG2x:HLA-E interaction has provided insight into the effect that specific peptides presented via HLA-E can have on the modulation of NK cell function. The majority of peptides can interact with both NKG2C and its inhibitory counterpart NKG2A, thus having the potential to both activate and inhibit NK cell function, respectively. However, a subset of peptides can selectively activate via NKG2C [26]. The specific peptide presented, in combination with the inflammatory signals, control the expansion of the resulting adaptive NK cell pool, as HCMV peptides are differentially recognized by adaptive NK cells, allowing them to distinguish between distinct HCMV strains [27]. The HLA-E peptide with the highest affinity to NKG2C/CD94 is the nonamer peptide derived from HLA-G VMAPRTLFL and its recognition is sufficient to induce maximally functional adaptive NK cells relating to their IFNγ and TNFα response [27]. Adaptive NK cells can discriminate between HLA-E bound peptides with a high level of specificity and co-culture with VMAPRTLFL-HLA-E beads enriches for FceRγ^−^ adaptive NK cells, a population that exhibits enhanced ADCC responses, particularly IFNγ secretion [28].

Adaptive NK cell proliferation appears to be restricted to HCMV-exposed individuals [29,30]. Recently, adaptive NK cells have been characterised in the peripheral blood, lungs and liver of HCMV-seropositive individuals [24,30,31]. Principal component analysis of epigenetic profiles has displayed striking similarities between adaptive NK cells and memory CD8 T cells [32]. In addition to NKG2C, adaptive NK cells also display high expression of the co-stimulatory molecule CD2, as well as downregulation of the inhibitory receptors Siglec-7 and CD161 [32,33]. Adaptive NK cells have several benefits over conventional/canonical NK cells. Loss of the signalling molecule FceRγ and increased phosphorylation of CD3ζ and ZAP70 confer enhanced ADCC responses [34]. In particular, adaptive NK cell functional responses are skewed towards IFNγ production, attributed to epigenetic modification of the *IFNG* locus [32]. Metabolic reprogramming results in an elevated bioenergetic profile, with heightened glycolysis and OXPHOS accompanying higher mitochondrial membrane potential compared to conventional NK cells [35]. Adaptive NK cells display very high expression of single self-KIR, giving them an enhanced capacity to recognise “missing self” and thus higher sensitivity to target cells [36]. Regarding their immunoregulatory role, adaptive NK cells display reduced bystander killing of activated T cells, thus not hampering the T cell arm of immunity [37]. Adaptive NK cells have been shown to be inherently resistant to suppression by myeloid-derived suppressor cells [38]. It is important to note that the adaptive NK cell pool is highly heterogeneous, reflecting individual, donor-specific diversity, as well as within-donor diversity, particularly relating to differences in receptor repertoire and maturation stage. Substantial clonal expansion and clonotype persistence of NKG2C^+^ memory NK cells in HCMV^+^ individuals have been demonstrated in a single-cell multiomic map of human NK cells from HCMV^+^ and HCMV^−^ individuals, resembling typical adaptive immune cell hallmarks [39]. A study carried out by the Romagnani group described pronounced epigenetic remodelling of the NKG2C^+^ NK cell pool imposed by HCMV, characterised by an inflammatory memory footprint enriched in AP1 transcription factors. They proposed clonal inheritance of epigenetic traits as a mechanism of accumulation of layers of heterogeneity [39]. These findings indicate that adaptive NK cells are superior to canonical NK cells, both functionally and in their capacity to withstand hypoxic and nutrient-depleted environments, making them an attractive target for immunotherapies.

Although human adaptive NK cells have been most extensively characterised in the context of HCMV infection, adaptive NK cells may play a role in controlling other viral infections. However, the high prevalence of HCMV infection has complicated analysis of other bona fide pathogen-specific NK cell responses. Recent studies have revealed a population of adaptive NK cells in hantavirus [40], chikungunya [41] and HIV [42,43,44,45], and SARS-CoV-2 [46,47] infections, but proliferation was partly driven by a coexisting HCMV infection. Preferential proliferation of the NK cell population has also been reported in other viral infections, such as Epstein–Barr virus (EBV) and hepatitis B virus (HBV) [48,49]. Recent research in HBV infection and HIV/HBV co-infection has shown that adaptive NK cells inversely correlate with HBV RNA levels, a surrogate marker for HBV intra-hepatic viral activity, and HBV surface antigen (HBsAg) levels, which impairs NK and T cell function, suggesting that adaptive NK cells play a role in controlling HBV infection [50].

A mechanistic study elucidated that human NK cell virus-specific memory developed upon exposure to both influenza and HIV, which was largely dependent on the NKG2C/HLA-E axis [51]. Upregulation of α4β7, KLRG1 and NKG2C were identified as phenotypic biomarkers to track influenza and HIV-specific NK cells [51]. The NKG2/HLA-E axis was shown to be responsible for the Gag- and Env- specific NK cells responses from the spleens and livers of SIV-infected rhesus macaques [52]. The difficulty in distinguishing NKG2C from other NKG2 family molecules in non-human primates has posed an obstacle to the delineation of the axis at play. Memory NKG2C^+^ NK cells in non-human primates were first identified by expression of the *KLRC2* gene, with these populations undergoing functional repertoire diversification upon rhCMV and SIV infections [53]. These findings suggest that, in addition to CMV infection, NK cells carry out memory responses against other pathogens, and further investigation would be required to dissect the molecular mechanisms at play in antigen-specific NK cells.

The well-documented association between CMV antigens, murine Ly49H and human NKG2C has provided a valuable case study for understanding how NK cells can mount an adaptive response. Indeed, adaptive NK cells may develop independent of Ly49H and NKG2C. In a mouse model, the MCMV immunoevasin m04 forms a complex with MHC-I and induces the DAP-12-associated activating receptors Ly49P and Ly49L [54,55]. Intriguingly, Ly49L^+^ NK cells were preferentially selected to expand clonally and provide protection in a MCMV-infected mouse model [54]. Apart from DAP-12, ITAM-containing adaptors CD3ζ and FceRγ can mediate signalling via NK cell receptors, such as activating Ly49 receptors, Fc receptor, NKp46 and NK1.1 [56]. Recognition of the MCMV immunoevasin m12 can be mediated by the activating receptor NK1.1 [57]. The emergence of redundant human adaptive NK cell subsets was observed in HCMV^+^ individuals carrying a homozygous deletion of the NKG2C-encoded gene (*KLRC2*−/−). Adaptive NK cells in NKG2C^−/−^ donors shared functional attributes with HCMV-induced NKC2C^+^ adaptive NK cells and were independent of activating KIRs. In addition, this study unravelled a critical role of CD2 in antibody-dependent responses by NKG2C^+^ and NKG2C^−^ adaptive NK cells [58]. HCMV infection can drive the expansion of CD56^dim^NKG2A^−^KIR^+^ NK cells, even in the absence of NKG2C expression in patients transplanted with NKG2C^−/−^ umbilical cord blood, where the activating KIRs trigger NK cell cytotoxicity, degranulation and cytokine secretion [59]. Further insight into this interaction was provided by a study that revealed that HCMV-driven modulation of HLA-C is required for KIR2DS1-mediated NK cell activation, considering that KIR2DS1 interacts with C2 group HLA-C [60]. These data suggest a role for activating KIRs in HCMV-driven NK cell reconstitution, maturation and effector function, which can be particularly relevant in the control of viral infections post--transplantation.

In addition to viral infection, bacterial infection may also confer antigen-specific memory-like features in NK cells in an NKG2C-independent manner. *Burkholderia pseudomallei*-specific NK cell responses were restricted to a pool of CD160^+^ NK cell population with memory-like features in humans. Frequencies of CD160^+^ NK cells with heightened responses upon antigen restimulation remain elevated for a prolonged period of time after infection. Intriguingly, elevated percentages of NKG2C^+^CD57^+^ NK cells were not detected following infection, further indicating that this NK cell pool is not a unique feature of *B. pseudomallei* infection [61,62]. These studies indicate that various NK-cell-activating receptors could induce antigen-specific clonal expansion and differentiation, suggesting a critical widow of opportunity for identifying mechanistic induction of NK cell memory and other populations of antigen-specific memory NK cells in addition to CMV-driven adaptive NKG2C^+^CD57^+^ NK cells.

## 3. Non-Antigen-Specific Memory-like NK Cells

Cytokine-induced memory-like (CIML) NK cells are induced after a brief period of exposure to the cytokine cocktail IL12/15/18 followed by a differentiation period in low-dose IL-15. The generation of these cells was first observed in mouse NK cells activated ex vivo with the triple cytokine cocktail, where these NK cells persisted for several weeks after being transferred back into the mice and showed enhanced IFNγ secretion after restimulation ex vivo [63]. CIML-NK cells have characteristics of immunological memory, but unlike adaptive NK cells, they have not shown responses to specific antigens.

Similar observations have been made utilising human NK cells, with first reports showing enhanced cytotoxicity and IFNγ secretion upon cytokine restimulation compared to control NK cells, both against cancer cell lines and primary leukemic blasts [64,65]. After IL12/15/18 stimulation, the phenotype of CIML-NK cells includes changes in the expression of several NK cell markers, and these cells are characterised by their surface marker expression as CD25^hi^CD94^hi^NKG2A^hi^CD69^hi^NKp46^hi^ [63,64,65]. While thought to be a homogenous population, recent CITEseq evidence has revealed the emergence of two populations enriched in bulk memory-like NK cells, termed enriched memory-like (eML). These cells are characterised by the expression of markers CD117^−^CD57^−^NKG2A^+^CD39^+^. These clusters have reduced expression of genes associated with maturation (*FGFBP2* and *SPON2*) and increased expression of genes associated with trafficking and adhesion (*CX3CR1*, *S1PR1*, *S1PR5* and *ICAM2*), together with enhanced cytokine secretion and cytotoxic capacity. Interestingly, eML NK cells are detected at baseline in the peripheral blood of donors and are significantly induced ex vivo after IL12/15/18 exposure and a 7-day differentiation process. The appearance of eML subpopulations has been confirmed in cancer patients receiving ML NK cell therapy up to 60 days after patient infusion [66].

A potential mechanism involved in this enhanced function is through the increased expression of CD25 and increased STAT5 phosphorylation that CIML-NK cells exhibit over time, allowing these cells to respond to picomolar concentrations of IL-2, which translates to enhanced functional responses [67]. Epigenetic imprinting, akin to adaptive NK cells, has been observed in CIML-NK cells, elucidating their augmented functionality. Specifically, demethylation of the CNS1 region of the *IFNG* gene has been identified as a critical contributor to the stabilization of the IFNγ-producing phenotype [66,68,69]. Exciting new evidence has uncovered a detailed explanation on the epigenetic and transcriptomic changes driving the generation of CIML-NK cells. ATACseq analysis identified memory-like (ML) initiation, resulting in 6875 differentially accessible regions (DARs), including IFNG, IL2RA, SOCS1 and TF downstream of cytokine activation. Of those, 785 DARs persisted, and 312 new ones emerged after allowing for 7-day differentiation into ML, explaining the sequential process by which CIML-NK cells acquire memory features [66].

Another feature of memory NK cells is their metabolic profile. NK cells modify their metabolism to support different processes and, similar to adaptive NK cells, CIML-NK metabolism has been studied after IL12/15/18 stimulation. CIML-NK cells increase their expression of nutrient transporters like the expression of the heavy subunit of multiple heterodimeric amino acid transporters CD98, and the glucose transporters GLUT1. This correlated with an enhancement in their glycolytic rate after 7 days in culture. Interestingly, while cytokine-activated NK cells upregulated OXPHOS and spare respiratory capacity (SRC), these differences were not observed in CIML-NK after 7 days in culture [70]. Despite this increase in glycolysis, preliminary evidence shows that CIML-NK cells might accumulate unfit mitochondria during the differentiation period. Mitochondrial potential was decreased in the CD56^dim^ population after IL12/15/18 stimulation [71]. It has been shown that upon IL12/15/18 stimulation, NK cells incremented mitochondrial superoxide levels measured by MitoSOX staining [72]. This preliminary evidence raises the possibility that exposure of CIML-NK cells to high cytokine doses maximises their metabolic capacity, potentially reducing their metabolic fitness, an aspect that should be considered when designing therapeutic strategies.

NK cells with characteristics of immunological memory have also been described in the context of tumour-priming. NK cells are primed upon exposure to certain cancer cell lines, and they gain the ability to respond to cell lines they did not respond to previously [73,74,75,76]. There is conflicting data on whether these NK cells show signs of antigen specificity, Pal and colleagues showed that tumour-primed NK cells exhibited enhanced cytotoxicity exclusively towards the cells they were primed with, but extensive research by the Lowdell group has shown that these NK cells can kill a variety of targets, resembling CIML-NK cells [74,75,76,77]. These NK cells upregulate CD69, CD25, CD57 and CD132, and share phenotypic and proteomic clusters with CIML-NK cells [77]. The generation of these tumour-memory NK cells is driven by CD2 ligating CD15 on the tumour cell [78]. Tumour-primed NK cells show expansion of metacluster “3”, which is characterized by high expression of CD2, further confirming the importance of the CD2-CD15 interaction in tumour-priming [77]; thus, it appears that memory induced by tumour-priming share a dependence on CD2 ligation with memory generated during CMV infection [58,77,78,79,80].

**Figure 2:**
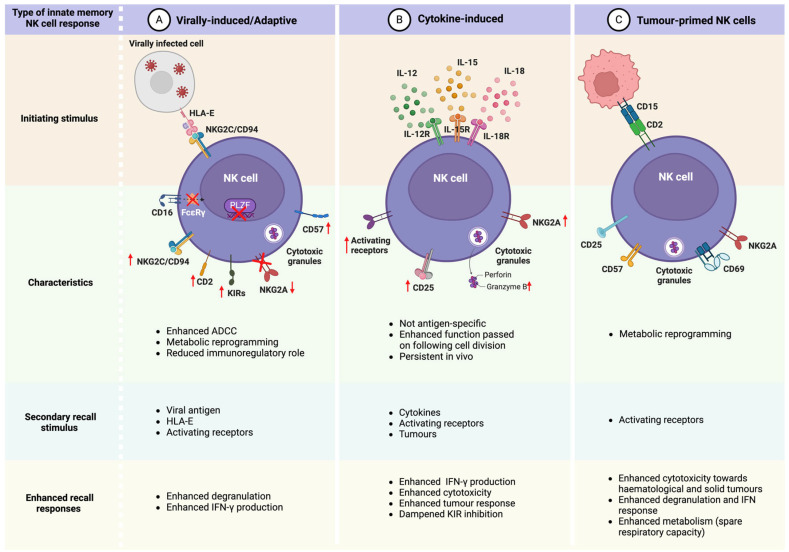
Phenotypic and functional characteristics of adaptive, cytokine-induced and tumour-primed NK cells.

Owing to their attributes, CIML and adaptive NK cells are superior to conventional NK cells and their differences relating to receptor repertoire and functional responses allow for their utilization in different clinical contexts depending on the purpose of the intended therapy. Figure 2 shows an overview of these differences and similarities.

## 4. Clinical Application of Memory NK Cells

Insights into NK cell biology gained from viral infections have driven efforts to translate findings in clinical settings, informing vaccination and adoptive cell therapies. While the majority of NK-cell-based therapies rely on conventional NK cell properties, harnessing the superiority of memory NK cells over canonical NK cells provides an avenue for designing more effective immunotherapies.

While current vaccination strategies target the ability of T and B lymphocytes to form long-lasting specific memory, NK cell memory can be harnessed for the same reason. The enhanced capacity of adaptive NK cells in controlling HCMV infection provides a rationale for the development of vaccines that induce adaptive NK cell expansion. Vaccination is an essential preventive strategy to lessen the impact of infection. The expansion of effector and memory lymphocytes and/or the levels of neutralising antibodies are typical quantitative indicators of protection. The initial evidence for NK-cell-mediated recall responses was found in contact hypersensitivity using B and T-cell-deficient mice. Chemical hapten sensitisation led to antigen-specific NK-cell-mediated recall responses at the rechallenge site [14]. Another important consideration in advancing NK cell memory-induced vaccines was raised in the finding that CXCR6^+^ hepatic NK cells can mount antigen-specific recall responses [15,81]. Challenging B and T-cell-deficient mice with viral-like particles from HIV, influenza and VSV also triggered enhanced recall responses, underscoring the potential applicability for vaccination strategies. However, the receptors and viral ligands responsible for recognition have not yet been identified [15].

Growing evidence supports the contribution of NK cells in both effector and memory phases in response to various vaccines [82,83,84]. NK cells from vaccinated individuals exhibited increased influenza-specific IFNγ response to influenza antigen upon restimulation in vitro and high cytotoxic response to autologous HBsAg-pulsed monocyte-derived DCs [49,85]. Likewise, BCG bacillus Calmette–Guérin (BCG) revaccination induced high production of NK-cell-derived IFNγ upon BCG restimulation [86]. Antigen-specific memory NK cell responses were also described in non-human primates immunised with Simian immunodeficiency virus (SIV) and HIV envelope protein (HIV-env)-preloaded DC immunisation, although currently there is no available vaccine for HIV infection [52]. Increased NK cell activity and expansion of NKG2C^+^ NK cells in CMV seropositive donors were also observed upon re-exposure to the receptor binding domain of the SARS-CoV-2 Spike protein in combination with IL-12 and IL-18 nine months post-vaccination [87]. However, the molecular mechanisms of these potentially antigen-specific NK cells remain unknown. SARS-CoV-2 vaccination in people living with HIV induced expansion of a more differentiated, adaptive NK cell population, exhibiting enhanced antibody-dependent NK cell responses. The accumulation of adaptive NK cells associated with the magnitude of cellular and humoral responses [47], highlighting the benefit of the incorporation of vaccines that induce memory NK cells in further boosting vaccine-induced adaptive responses. Similarly, seasonal influenza vaccination generated CIML NK cells in a cohort of 52 previously unvaccinated individuals, with these cells displaying enhanced IFNγ responses to cytokine stimulation for months post-vaccination [88]. Vaccination can be utilized to preferentially expand NK cells with memory features and harness their Fc-dependent function and enhanced effector function.

Owing to their specific features, NK cells are considered as one promising target for immunotherapy. Attempts are now being made to identify antigen-specific memory NK cells for adoptive cell therapy in the fight against viral disease. In the absence of a gold standard treatment for COVID-19 disease, and despite the success of SARS-CoV-2 vaccines, several clinical trials have been initiated to ameliorate the outcome of severe disease. Cell therapy, which has demonstrated therapeutic efficacy against other viral infections, is becoming an appealing alternative for treating patients with COVID-19 [89]. Several clinical trials are employing NK-cell-based products to combat SARS-CoV-2 infection [90]. One study revealed that the presence of a robust NKG2C^+^CD57^+^ NK cell subset led to a heightened response against SARS-CoV-2 peptides in convalescent individuals, highlighting the importance of virus-specific NK cells as one of the criteria to select donors to generate off-the-shelf drugs for adoptive cell therapy against SARS-CoV-2 infection in clinical trials (NCT04578210) [91]. In addition, expanded clinical-grade NK cells exhibited stronger protection against refractory HCMV infection in a cohort of 20 post-allogenic haematopoietic stem cell transplantation patients, where CMV reactivation presents a common complication and is associated with higher mortality [92]. This highlights the potential role of adaptive NK cells in controlling viral infection, and although promising, this remains a relatively understudied area of research.

Insights provided from harnessing memory NK cells in the setting of viral infection have been extended to haematological and solid tumours. Currently, approximately 220 open studies are registered at ClinicalTrials.gov investigating the potential efficacy of NK cells as immunotherapy. In the context of haematological malignancies FATE-NK100, an allogeneic donor-derived adaptive NK cell immunotherapy, has been investigated against refractory or relapsed acute myelogenous leukaemia (AML). In the VOYAGE clinical trial (NCT03050216), all patients were reported to have benefitted from the treatment by reaching a morphologic leukaemia-free state by day 14 after treatment [93]. Safety, persistence and elevated function of adaptive NK cell (FATE-NK100) intraperitoneal infusions from CMV seropositive donors in 12 patients with refractory ovarian cancer were reported in the APOLLO clinical trial (NCT03213964) [94]. The enhanced capacity of adaptive NK cells for ADCC can further enhance responses to monoclonal antibody therapies. To that end, FATE-NK100 is being investigated in combination with two monoclonal antibodies: the anti-EGFR monoclonal antibody cetuximab in patients with advanced colorectal cancer and head and neck squamous cell cancer or the anti-HER2 monoclonal antibody trastuzumab in patients with HER2^+^ advanced breast, gastric or other solid cancers (DIMENSION trial NCT03319459) (from https://clinicaltrials.gov, accessed on 1 November 2024).

The versatility of CIML-NK cells is highlighted by recent attempts to engineer these populations. CIML-NK cells have been combined with CAR against AML neoepitopes enhancing their anti-tumour responses and reducing off-target effects [95]. CIML NK cells have shown their safety and efficacy in the treatment of acute myeloid leukaemia patients [65,96] and are currently being tested in several clinical trials (NCT01898793, NCT02782546, NCT03068819, NCT04024761, NCT04290546, NCT04354025 and NCT04634435 (from https://clinicaltrials.gov, accessed on 11 July 2024)). Adoptive transfer of CIML NK cells has produced impressive results, with complete clinical remissions observed in relapse/refractory acute myeloid leukaemia [65]. Similarly, the adoptive transfer of expanded adaptive memory-like NK cells has proven successful in the context of haematological malignancies. Expansion of single self-KIR^+^NKG2C^+^ adaptive NK cells from select superdonors has displayed strong alloreactivity against HLA-mismatched acute myeloid leukaemia [97], revealing an opportunity for off-the-shelf, non-engineered and highly selective NK cell therapies.

**Figure 3:**
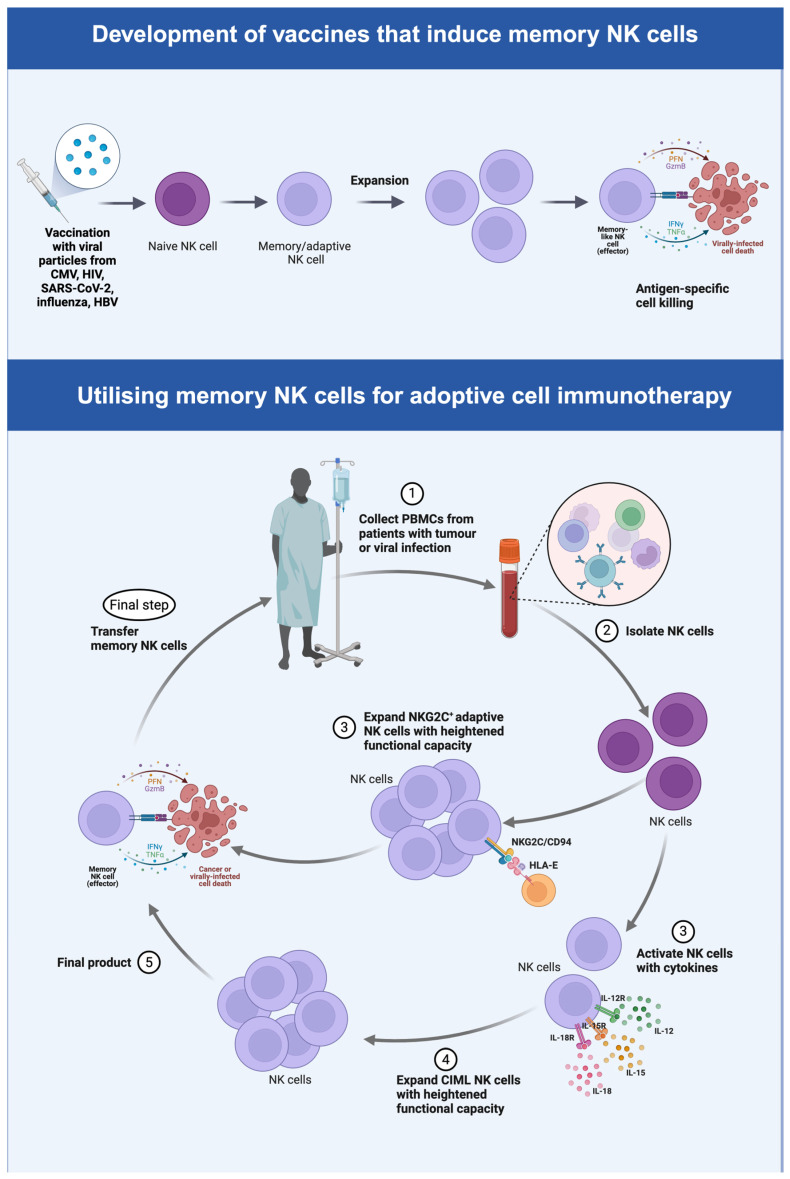
Memory NK cell application in vaccination and adoptive cell therapy strategies. Vaccination with viral particles can induce memory NK cells with enhanced effector function. Memory NK cells can be induced and expanded ex vivo and infused in patients with viral infection or tumour as adoptive cell therapy.

Figure 3 below summarises the principles behind the incorporation of memory NK cells in vaccination and adoptive cell therapy strategies against viral infection and tumour.

## 5. Concluding Remarks

Research on NK cell properties beyond their classical innate characterisation has provided insights into NK cell subsets with distinct phenotypic and functional attributes, such as memory NK cells. The superior qualities of these NK cell subsets to conventional NK cells, relating to their enhanced functionality, persistence, clonal expansion and metabolic reprogramming, can be harnessed to design more effective immunotherapies in the context of viral infection and malignancy. While this is a relatively new area of research, results from translational research and clinical trials are promising and highlight the benefit of incorporating memory NK cells in therapeutic strategies against viral infection and malignancy.
